# Splenic Abscess in an Adolescent due to *Salmonella enterica* Conservatively Treated with Ultrasound-Guided Fine-Needle Aspiration

**DOI:** 10.1155/2023/8802760

**Published:** 2023-03-13

**Authors:** Anne Sofie Steinbring Jørgensen, Ivan Arsic, Niels Pedersen, Lena Hagelskjær Kristensen, Michael F. Nielsen, Thomas Balslev

**Affiliations:** ^1^Department of Paediatrics, Viborg Regional Hospital, Viborg, Denmark; ^2^Department of Radiology, Viborg Regional Hospital, Viborg, Denmark; ^3^Department of Internal Medicine, Viborg Regional Hospital, Viborg, Denmark; ^4^Department of Surgery, University Hospital of Southern Denmark, Odense, Denmark

## Abstract

**Background:**

Splenic abscesses are rare and potentially fatal. Diagnosis is often delayed due to vague symptoms, and laboratory findings are varying and often nonspecific. Ultrasound and computed tomography have a high sensitivity in detecting splenic abscesses. Splenectomy was previously considered the gold standard for treatment, but in recent years, a shift has been seen towards a more conservative approach, i.e., ultrasound-guided aspiration or drainage in combination with adequate antibiotics in selected cases. *Case Report*. A previously healthy adolescent complained of left-sided chest pain, pain in the left clavicular region for three weeks, and recent fever. Ultrasound and computed tomography demonstrated an intrasplenic abscess. The patient was successfully treated with two percutaneous fine-needle punctures and adequate antibiotics for six weeks. *Salmonella enterica* serotype Poona was grown from the aspirate. At one-year follow-up, the patient remained healthy without signs of recurrence.

**Conclusion:**

The present case report demonstrates that ultrasound-guided aspiration and subsequent treatment with antibiotics may be an effective alternative to splenectomy in patients with a splenic abscess.

## 1. Introduction

Splenic abscess is a rare and potentially life-threatening disease. It can occur in immunocompetent children, but is usually seen in adults (mean age group 54.1 ± 14.1 years [1]), with a reported incidence of 0.14–0.7% [2] based on autopsies in adults. It is thought to be less frequent in the paediatric population [3]. The diagnosis is often delayed [4] due to nonspecific symptoms. The triad of fever, left-upper abdominal pain, and palpable left-upper abdominal swelling is observed in up to one-third of patients [5]. Most patients present with fever [6]. Patients often suffer from comorbidities (e.g., immunodeficiency, haemoglobinopathies, malignancy, and diabetes mellitus), and in some cases, infection is due to haematogenous spread from other sites or secondary to trauma or splenic infarction [7].

The microbiological agents causing splenic abscesses differ around the world [1]. Microbiological identification is therefore essential to target antibiotic treatment, and material for culture may be achieved by ultrasound (US)-guided aspiration. US plays a valuable role in the diagnosis of splenic abscess, with a sensitivity of 90–98.8% [1, 8] in experienced hands, but the diagnosis should be confirmed by computed tomography (CT), which has a sensitivity of 95–100% [3]. The optimal treatment option remains unclear. In recent years, there has been a shift from splenectomy towards a more conservative approach with use of percutaneous aspiration or drainage, combined with antibiotic treatment [9, 10]. Most data are based on adults, and there are no definite guidelines for managing splenic abscesses in children.

## 2. Case Presentation

A 15-year-old adolescent with no previous medical history was admitted to the Paediatric Emergency Department due to a suspicion of pleuritis. His symptoms began five weeks earlier, two days after returning from Ghana, where he had spent two weeks. He had obtained all the recommended immunisations according to national guidelines, had taken relevant hygienic precautions, and received adequate antimalarial prophylaxis. In Denmark, he developed high fever, diffuse abdominal pain, nausea, vomiting, and nonbloody watery diarrhoea. Symptoms persisted for one week and disappeared without medical treatment. One week later, he developed a dull pain in the left side of his chest that worsened during deep inspiration. Symptoms intensified over a three-week period, and when he developed fever, he was referred to the Emergency Department.

Upon admission, his temperature was 38.3°C, heart rate 107 beats/minute, respiratory frequency 14/minute, blood pressure 117/71 mmHg, and capillary filling time <2 seconds. Physical examination revealed a tender filling below the left curvature. Tenderness was also found in the soft tissue above and below his left clavicle. Otherwise, his clinical examination was normal. Blood analysis revealed elevated CRP (58.4 mg/L), but the normal white cell count (8.9 × 10^9^/L) and normal levels of platelets, haemoglobin, and organ markers. The chest X-ray was normal.

**Figure 1:**
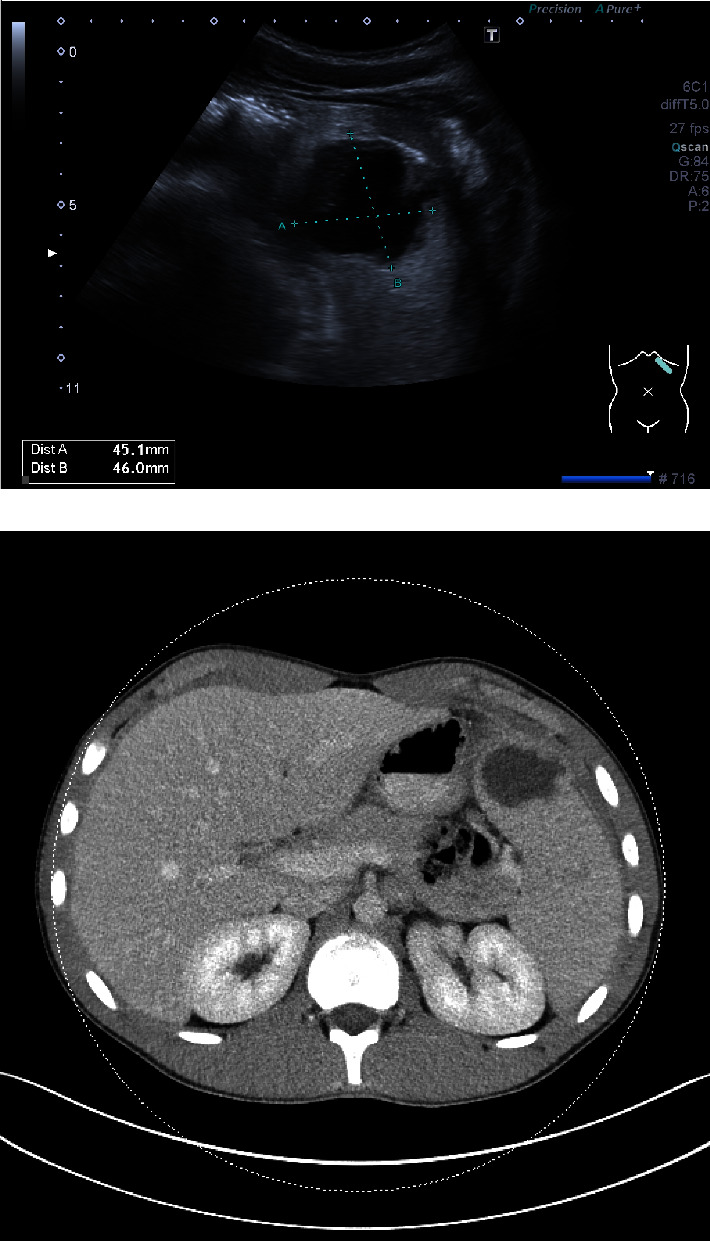
(a) US image of the intrasplenic cystic process with thick wall that proved to be an abscess. (b) Image of the initial CT scan prior to the 1^st^ puncture illustrating splenomegaly and the site of the abscess next to the abdominal wall.

US revealed splenomegaly (17 cm) and a hypoechoic cystic process, suspected to be an abscess (Figure 1(a)). A low-dose CT scan with intravenous and oral contrast (Visipaque) confirmed a 32 × 45 × 40 mm thick-walled, unilocular abscess anteriorly in the spleen, with an adjacent inflammatory reaction in the abdominal wall (Figure 1(b)), and a small amount of ascites. Distance between skin and abscess was 30 mm. An US-guided fine-needle aspiration was performed. The anterior placement of the abscess made it possible to perform the procedure using a subcostal approach and thereby minimizing the risk of contact with the diaphragm or the pulmonary pleurae. After administration of local anaesthesia (Xylocaine), a Ø 0.9 mm needle was used for aspiration. 40 mL purulent material was aspirated and the cavity was flushed twice with 10 mL saline. US confirmed a subsequent collapse of the abscess cavity. Antibiotics were not prescribed initially.

Twelve hours later, the patient experienced chills and fever spiked up to 40.2°C. Blood was drawn for culture, and he was started on IV ceftriaxone 50 mg/kg daily. The following day, the fever persisted and the left clavicular pain returned. A repeat US three days later confirmed recurrence of the abscess. Using the same method as initially, 10 mL of the purulent material was aspirated by a fine needle and the cavity was flushed with saline. US confirmed that the cavity had collapsed. The pain in the left clavicular region disappeared. From the initial aspirate of the abscess grew a monoculture of *Salmonella enterica* subsp. *enterica serovar* Poona. Three subsequent sets of stool samples and four sets of blood cultures did not reveal any growth. US was performed every two–four days for the first 14 days and hereafter monthly. After 16 days of ceftriaxone intravenously, the CRP had normalised and the patient was asymptomatic. He completed four weeks of antibiotic therapy with oral ciprofloxacin 500 mg × 2 daily.

After two months, the initial splenomegaly had diminished to 14.2 cm, and the abscess could no longer be visualised by US. The patient was monitored for one year with US every three to six months. The spleen remained mildly enlarged. All immunological parameters remained normal. No detectable signs of haematological, systemic, malignant, or concurrent infectious disease were detected. At one-year follow-up, which involved a routine clinical examination, the patient was well and he had resumed elite sports without restrictions.

## 3. Discussion

The patient's history is illustrative of an immunocompetent adolescent with a solitary splenic abscess presenting with vague symptoms. On admission, the patient presented with fever, upper-left quadrant pain, and raised CRP. Noteworthy, the patient did not present with leucocytosis, which is seen in up to 70.1% of patients with a splenic abscess [1, 3]. The patient presented Kehr's sign [11], a referred pain to the ipsilateral clavicular region due to subdiaphragmatic irritation of the phrenic nerve deriving from the abscess. The pain in the left clavicular region disappeared after the first aspiration, but returned with the recurrence of the abscess illustrating Kehr's sign.

The optimal treatment for splenic abscess remains unclear, especially in the paediatric population. Treatment options include percutaneous aspiration, percutaneous catheter drainage, open drainage, and splenectomy (partial or total; open or laparoscopic) [12] alone or in various combinations. Compared to open splenectomy, conservative treatment with US-guided aspiration in combination with antibiotics is a minimally invasive procedure associated with fewer side effects and reduced hospitalisation [3, 9, 10, 13]. Percutaneous abscess aspiration instead of splenectomy eliminates the need for lifelong vaccination and precautions against encapsulated bacteria, and especially in children, it reduces the risk for overwhelming postsplenectomy infection [14].

The treatment of this patient involved a close collaboration of the paediatric, radiological, microbiological, and surgical departments. We chose percutaneous needle puncture in our case due to the patient's unremarkable clinical status, the size of the abscess (maximal length 45 mm), and the anterior location, making it readily accessible to percutaneous puncture.

It is not uncommon for abscesses to recur [9]. Based on clinical and ultrasonic worsening, we decided to perform the 2^nd^ needle puncture. Splenic preservation was a high priority. If the 2^nd^ needle puncture had been unsuccessful or the patient's clinical status had further aggravated, we would have considered splenectomy.

## 4. Conclusion

In an adolescent with a solitary splenic abscess, US-guided fine-needle aspiration in combination with broad-spectrum antibiotic treatment is feasible and can be considered as an alternative to splenectomy, provided the patient's clinical condition is favourable.
